# Ethics, pandemic and environment; looking at the future of low middle income countries

**DOI:** 10.1186/s12939-020-01296-z

**Published:** 2020-10-15

**Authors:** Faouzia Tanveer, Ali Talha Khalil, Muhammad Ali, Zabta Khan Shinwari

**Affiliations:** 1 Department of Biotechnology, Quaid-i-Azam University, Islamabad, Pakistan; 2 Department of Pathology, Lady Reading Hospital (MTI), Peshawar, Pakistan; 3 Pakistan Academy of Sciences, Islamabad, Pakistan; 4 Department of Plant Sciences, Quaid-i-Azam University, Islamabad, Pakistan

**Keywords:** Coronavirus, Pandemic, Ethics, Environment, LMICs

## Abstract

COVID-19 which started in Wuhan, China and swiftly expanded geographically worldwide, including to Low to Middle Income Countries (LMICs). This in turn raised numerous ethical concerns in preparedness, knowledge sharing, intellectual property rights, environmental health together with the serious constraints regarding readiness of health care systems in LMICs to respond to this enormous public health crisis. From the restrictions on public freedom and burgeoning socio-economic impacts to the rationing of scarce medical resources, the spread of COVID-19 is an extraordinary ethical dilemma for resource constrained nations with less developed health and research systems. In the current crisis, scientific knowledge and technology has an important role to play in effective response. Emergency preparedness is a shared responsibility of all countries with a moral obligation to support each other. This review discusses the ethical concerns regarding the national capacities and response strategies in LMICs to deal with the COVID-19 pandemic as well as the deep link between the environment and the increasing risk of pandemics.

## Introduction

**Fig. 1:**
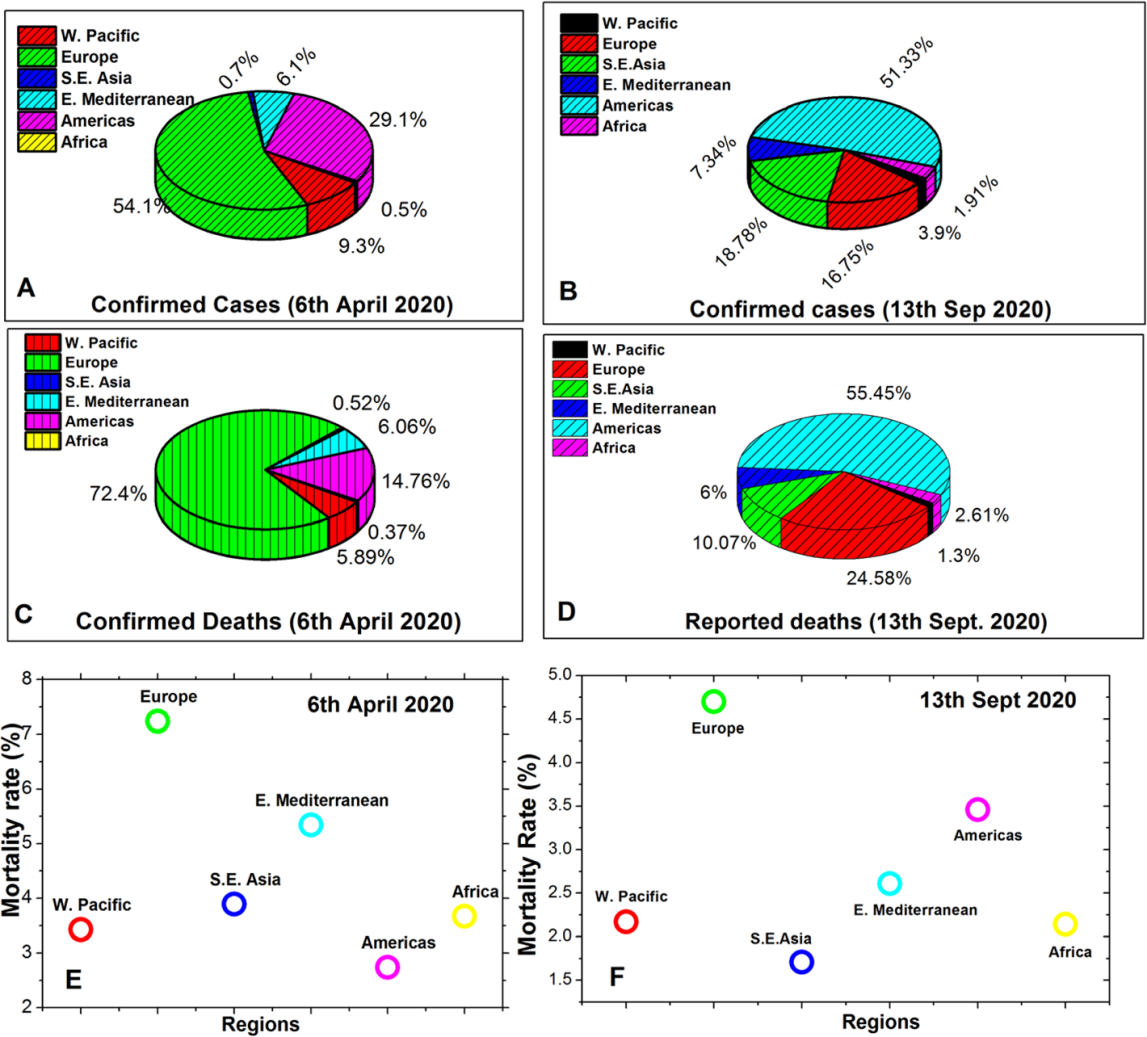
Summary of the situational report (Date taken from WHO; updated 6th April 2020 and 13th September 2020)

Like the previous outbreaks of coronaviruses i.e. Severe Acute Respiratory Syndrome (SARS) and Middle East Respiratory Syndrome (MERS), the ongoing pandemic COVID-19 has characterized that the infectious diseases represent a problem that does not recognize borders, race, ethnicity, religion, caste or any other status quo. Now known as “COVID-19”, “SARS-CoV-2”, “2019-nCoV”, the virus has already made a huge impact on a global scale [1] and changed human ways of thinking and characterizing the problem. COVID-19 is an issue beyond borders, thus necessitates a globally coherent, combined, inclusive and holistic approach which can help in the reduction of transmission and overall risk mitigation, which otherwise, is predicted to impact entire human race. According to the WHO situational report on 6th April, 2020, the total number of global cases surged up to 1,210,956 [2], with almost every country affected or threatened by the geographical expansion of SARS-CoV-2. The grand total of the total infections as of 13th Sept, 2020, is 28,637,952 with death toll of 917,417 [3]. A summary of the statistics taken from the WHO showing the data of 6th April 2020 and 13th September 2020 is indicated in inset Fig. 1 revealing the regional distribution of the SARS-CoV-2 cases and rate of mortalities. International regimes are on high alert to stop its spread, however, as far as the global scenario is concerned, countries and governments are clueless in stopping the expanding pandemic as not much is known about SARS-CoV-2, while left only with implementing nationwide lock downs and curfews which opened new economic fronts and social challenges. One of the major challenges is the intermittent psychological burden on segments of the society who have not been well versed with the scientific knowledge. Rumors and false information through social media brings enormous mental distress and singles out the need for responsible information sharing. Similarly, the deepened cultural norms that people find difficult to abandon in LMICs has created situations more favorable for transmission of SARS-CoV-2, with the religious fundamentalists also playing their part. Zoonotic origins of the coronaviruses and their circulation in the intermediate animal hosts presents another challenge of sustaining biodiversity and human-animal relationships. The primary reservoir of the SARS-CoV were bats while the intermediate source was civet cats that expanded across 29 countries in 2002–2003. MERS-CoV jumped to humans from camels and possessed an exceptional fatality rate of 35% in 2012. Now, SARS-CoV-2 has been proposed to jump to human beings from bats and pangolins [4, 5].

The ongoing pandemic has resulted in a situation in which the scale of emergency is similar to world war II (WW-II), requiring decisiveness and commitment [6]. In the developing and under developed regions, the risk management is extremely challenging because of the resource limitation as well as lack of basic health necessities and poor sanitation etc. [7, 8]. It is now established that the oral-fecal route of transmission of SARS-CoV-2 is also possible beside respiratory droplets and person to person contact which further multiply the complexities of SARS-CoV-2 for less advanced regions [9, 10]. Apart from the lack of resources and technology, negligence due to the lack of awareness presents a grim picture. COVID-19 has unleashed an enormous psychological burden that may have long term detrimental consequences.

COVID-19 has presented itself as a test case for the humanity in terms of global fraternity, decision making, technology and expertise sharing, rapid pandemic response mechanisms, stability, crises management and policy making. It is of paramount importance that the decisions regarding COVID-19 pandemic should be strictly governed by ethical and moral principles. A shared threat cannot be defeated without a combined response. Keeping in view the significance of the current situation, we have attempted to discuss various issues from the lens of ethics with special reference to the developing and under developing regions.

## General ethical concerns

COVID-19 pandemic is an unprecedented situation facing the world in current times, with large, unimaginable socio-economic impacts. In such a situation, pandemic preparedness and response efforts require careful analysis of core ethical values and principles with an informed and evidence-based decision making. The ethical aspects that require special consideration include the greater need for public engagement, disease surveillance, clinical research and novel experimental interventions. The moral obligations in relation to “duty to treat” and “duty to plan” must consider the rights of health care workers and affected communities. Moreover, necessary measures should be taken with respect to allocation of scarce resources, priority setting and social distancing [11]. The decision making process for outbreak preparedness planning involves a number of stakeholders including governments, NGOs, the military, commercial businesses, research funders, academic institutes, public health officials, researchers, ethicists, health care workers, volunteers, communities and families. All of them have different moral or legal obligations to fulfil [12].

### Ethics of preparedness

In a public health emergency, it becomes difficult to keep a balance between competing ethical principles i.e. need for necessary interventions in the interest of public health without compromising the public liberty. Measures that limit individual rights must be reasonable, proportionate, least-restrictive, impartial, non-discriminatory, and in accordance with national and international regulations [13]. When thoroughly implemented, home quarantine orders by government are legal and effective, as long as individual freedom and privacy is respected [14]. The principal of equal respect must be implied by decision makers when a lock down or quarantine is imposed on the public. Hereby restricting the public right to freedom is to be reciprocated by readily providing their basic needs, by ensuring effective risk communication through ethical and logical backing of this decision and giving easy access to latest information about the uncertain, ever changing risks [12].

Resource allocation should be ethical, transparent and based on scientific evidence. In this regard, the primary obligation is to protect front line health care workers as the entire health care systems depends on these individuals. Furthermore, public health measures should focus on prioritizing the provision of resources when and where required e.g. to the public in confined settings which are prone to rapid spread of disease (such as homeless shelters, prisons, and slum areas), to areas with localized outbreaks to control community transmission and to high-risk groups such as older people, people with co-morbidities and weekend immune systems [15]. Health equity i.e. equal health opportunities for all should be the focus of all health policies planned by the state actors to better prepare a country’s health system in the face of current pandemic or any health crisis that may come in future [16].

During a pandemic, issues of resource scarcity can be mitigated to a large extent if early public health interventions are introduced e.g. through social distancing which is crucial in reducing pressure on the health system. This is particularly important with regard to resource constrained settings such as those in Low to Middle Income Countries (LMICs). The failure to contain the spread at an early stage can severely constrain the health system’s capacity in these countries. Access to scarce resources which is considered reasonable in one country may be different in another such as in the case of developed and developing countries. Particularly in developing countries, the public should be well informed about decisions regarding allocation of limited resources with clear communication of proper justification to gain trust and avoid chaos [13].

Resource scarcity may also be encountered at the global level. Lower-income countries may face more scarcity than developed countries in countering COVID-19 spread. Hoarding of important medical supplies such as personal protective equipment and inaccessibility of vaccines and treatments when made available, should be discouraged by developed countries or the countries where they happen to be produced [15].

### Ethics in outbreak research and clinical practice

Ethical aspects must also be considered in COVID-19 pandemic research policy and practice. It is an ethical obligation to conduct research in infectious disease outbreaks needed to address pertinent research questions that arise during such a health crisis [17]. According to Nuffield Council on Bioethics, the core values of ethical research include helping reduce suffering, demonstrating equal moral respect for the communities involved and fairness in terms of benefit sharing. The ethical principle of helping reduce suffering provides the basis for prioritization of more valuable and much needed research during a public health emergency such as COVID-19 [12]. For example, conducting rapid review of research proposals becomes all the more important during a pandemic. However the decision of Ethics Review Committees (ERCs) should not be too hasty so as to avoid approval of mediocre or non-pertinent research at the same time ensuring a speedy review to facilitate important research. In these circumstances, Standard Operating Procedures (SOPs) could be introduced to form a multi-disciplinary sub-committee composed of members from ERC who could be immediately called in times of emergency to conduct rapid reviews [18]. To make the process more rapid, technological interventions should be encouraged. An ERC in a Chinese hospital used the video conference to review batches of research proposals. Moreover, these conferences were held more frequently during the corona virus pandemic than they normally did. The mean time between receiving the application and initial review decision was 2.13 days [19].

Ethical principle of fairness entails the equitable sharing of benefits and the burdens of research between different actors involved in research i.e. the participating community as well as the collaborating partners from low and high resourced settings. Similarly, the principle of equal respect emphasizes respectful relationships between researchers and the affected communities going through the emergency for meaningful community engagement. With respect to health care workers and researchers, the employers and the funders are responsible to make sure their needs are met as an equal moral obligation in exchange for their services [12]. Science and technology should be at the forefront of the outbreak research ranging from health sciences including risk assessment, risk management, vaccine development and modelling studies for improved data analysis to social sciences fighting discrimination/violence and promoting human rights [20]. In contrast to Research and Development (R&D) focused on medical care and treatment, less attention is given to the improvement of coordination in assessment and modelling studies on data generated during an outbreak. Integration of data analysis generated across disciplines is critical to provide support to decision makers during a pandemic in order to understand the course of the outbreak, the risk of its spread, and the potential effects of infection control measures [21]. This should be given due share in research practice during a pandemic.

Ethical standards also advocate the notion of “Duty to Care” and “Duty to Treat” by health care professionals during pandemics. Supporting arguments in relation to professional duty in the face of uncertainty and risk to life are guided by ethical principles of virtue, generosity and social utility [22, 23]. Besides, in dealing with this COVID-19 crisis, health care workers may have to take difficult decisions based on a utilitarian approach when faced with ethical dilemma of managing critical care resource allocation. Keeping in view the uncertainty surrounding this novel outbreak, rationing of resources might be required for a much longer time period and a far larger number of people. The response decision may require shifting from providing all the patients the maximum number of available resources to allocating minimal resources necessary for an individual’s survival. So that the additional resources are left out for others who may have an equal chance of a good outcome [24]. This is where governments and health care departments are obliged to guide and provide training to health care workers to handle difficult situations. Furthermore, ethical practice emphasizes the Duty to Plan where proactive planning by the public health leaders and health professionals to prepare beforehand can help reduce morbidity and mortality in a worst case scenario. The aim is to have a system in place across all levels of health care to maximize benefits to the community in the time of need [25].

## COVID-19: psychological burdens, pandemic to infodemic

Besides being an expanding pandemic, SARS-CoV-2 is accompanied by huge chunk of information floating through the social, electronic and print media making it the surge for authentic information and news much harder, as iterated by the WHO and UNICEF [26] . While people must rely on authentic data, the news spread through social media platforms often masks the original news/statistics. The tsunami of in-correct information and rumors has appeared as a major concern. The focus should be on awareness regarding SARS-CoV-2 and not on overburdening people with psychological distress which may lead our way to a psychological pandemic. One of the key steps to reduce the spread of misinformation is to automatically direct the users seeking information to WHO when keywords like coronavirus, COVID-19, pandemic etc. are searched on the online platforms. The only way in which traditional media will be helpful in fighting the expansion of the SARS-CoV-2 pandemic is through responsible reporting and sharing so that the information trickles down to common people. In pandemic of this global scale, media can be used as a source to mobilize communities to help the underprivileged segments of the society by keeping with the general safety protocols. Team of social media experts linked to the official sources can be helpful in diffusing correct information across the social media platforms. Evidence based information can be sought through the country specific official advisories and WHO. Limiting ones information resources can be helpful. Media giants must be adhered to strict norms of not to create panic but spread awareness.

## Environmental ethics, climate crises and COVID-19: preparing for the worst

**Fig. 2:**
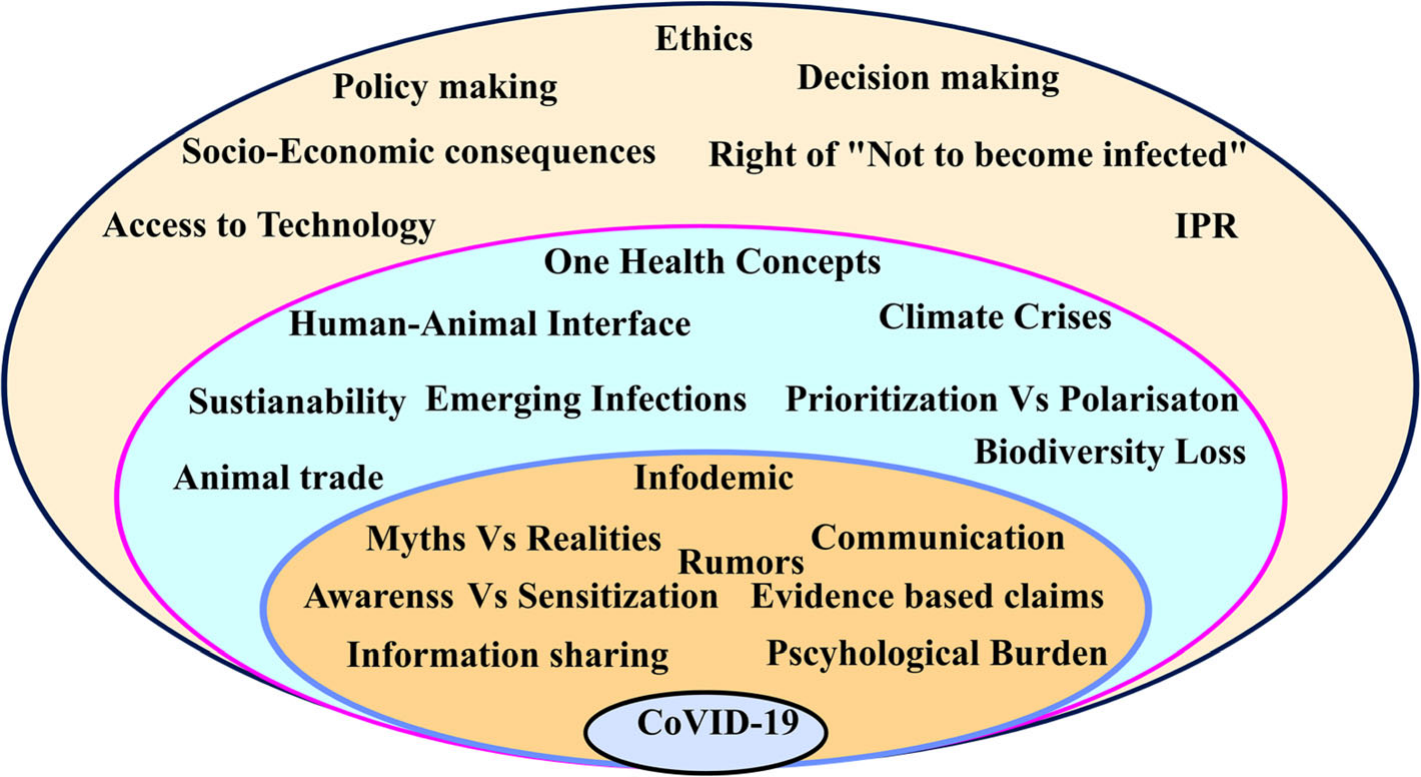
A holistic view of the ongoing pandemic of SARS-CoV-2

**Fig. 3:**
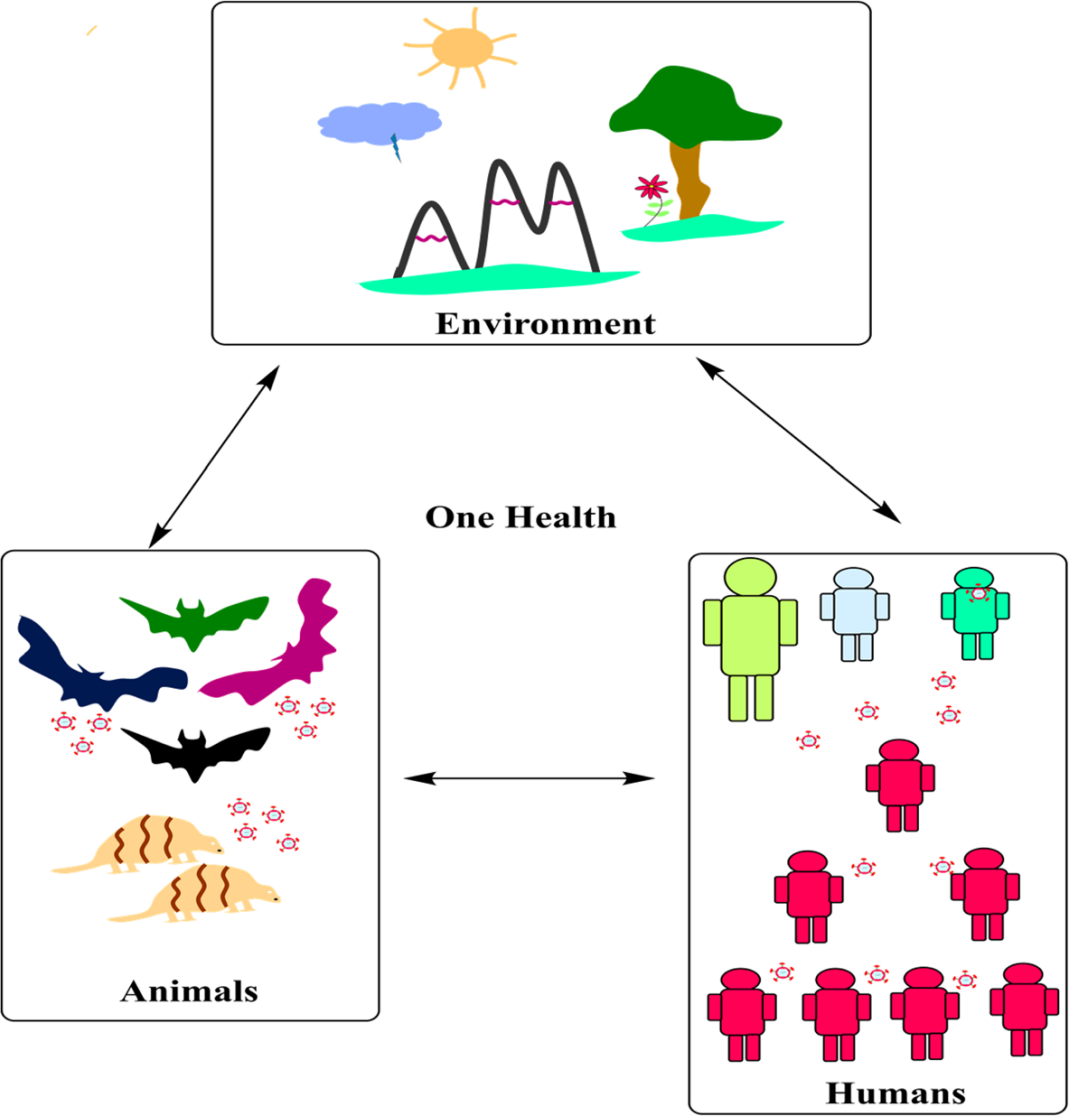
Inter dependence of the human, animal and environment - One Health Concept

COVID-19 pandemic is an example of complex threat to humanity from emerging and re-emerging pathogens and signifies the need for a holistic and integrated one health approach for reducing their risk [1]. One health approach is characterized by the inter dependence of human, animal and environmental health [27]. Both the animals and environment have a significant role in the emergence of infections with zoonotic origin in human population. Several factors like climate crises, increased travelling, population explosion, urbanization, deforestation, animal trade and rapidly evolving pathogens have further amplified the threat of emerging zoonosis. Due to evolutionary pressures and acquiring mutations, previously an animal pathogen, now gains the ability to cross the specie barrier, jumping and adapting to a new host i.e. human, which happened in case of SARS, MERS and now COVID- 19 [28, 29]. Circumstantial evidence suggest that the pandemic started in the seafood market which was a hotspot for buying and selling animals like bats, snakes, poultry etc. and provided sufficient human-animal interaction leading to spillover. Initial studies on the genome of SARS-CoV-2 reveals 96.11% similarity with bat coronaviruses leading to the conclusion that these viruses emerged from horseshoe bats [30]. Studies also revealed pangolins as one of the possible intermediate host [31]. These converging evidences signify the need for one health approach. Increasing demand for urbanization has led to human encroachment of more and more natural habitats, thereby, increasing exposure to novel exotic pathogens from the wild. A rapid consensus is building among the scientific community which infer the transition from Holocene era to Anthropocene era on the geologic time scale, in which human species are involved in changing the geology of the planet through anthropogenic activities [32]. As a consequence of plastic pollution, distribution of radioactive material across the planet, CO_2_ emissions, mining, deforestation and the sea level rise, the global ecosystem is becoming destabilized with time and threatens the animal species in the wild which may otherwise serve as a buffer between human and animals for harboring deadly infections. Extinction of megaflora and megafauna signifies the need of exclusive one health strategies to combat this ever expanding threat. The emerging diseases and climate crises cannot be separated and requires extensive research, funding and attention of the international leaders. Climate action cannot be shelved even in the pandemic as it is one of the tools for mankind to fight the emerging and re-emerging pathogens. Figure 2 indicates a holistic perspective of the SARS-CoV-2 pandemic while Fig. 3 illustrates the one health concept.

Adding more to the role of the environment, it is pertinent to mention that the developed countries are the major contributors towards the greenhouse gas emissions leading to global warming and climate change. This raises an ethical dilemma as most of the countries affected as a result of these changes are contributing negligible amount of Green House Gases (GHGs) but often become the adversely affected. The burden of responsibility regarding contributions to the climate change in relation to the pandemic needs significant discussions and dialogues.

## Ethical issues concerning COVID-19 outbreak: situational analysis in low to middle income countries (LMICs)

**Fig. 4:**
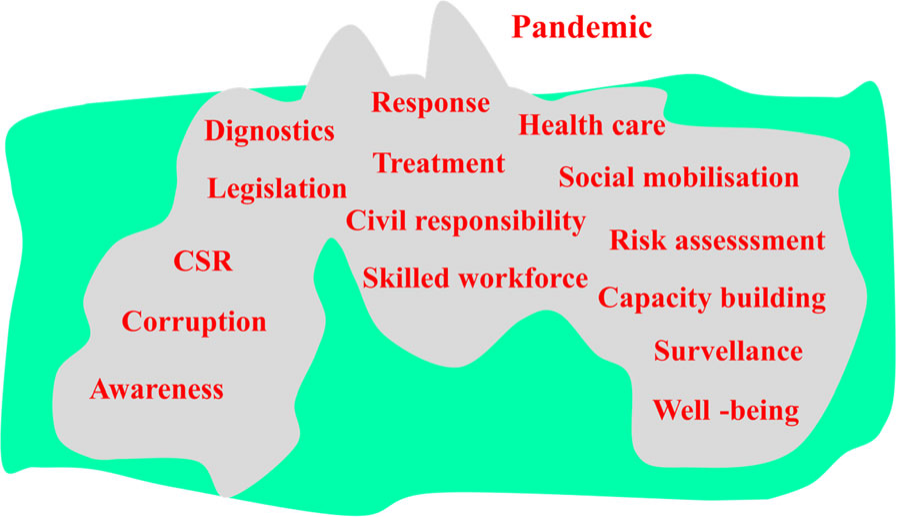
Health sector issues in LMICs

**Fig. 5:**
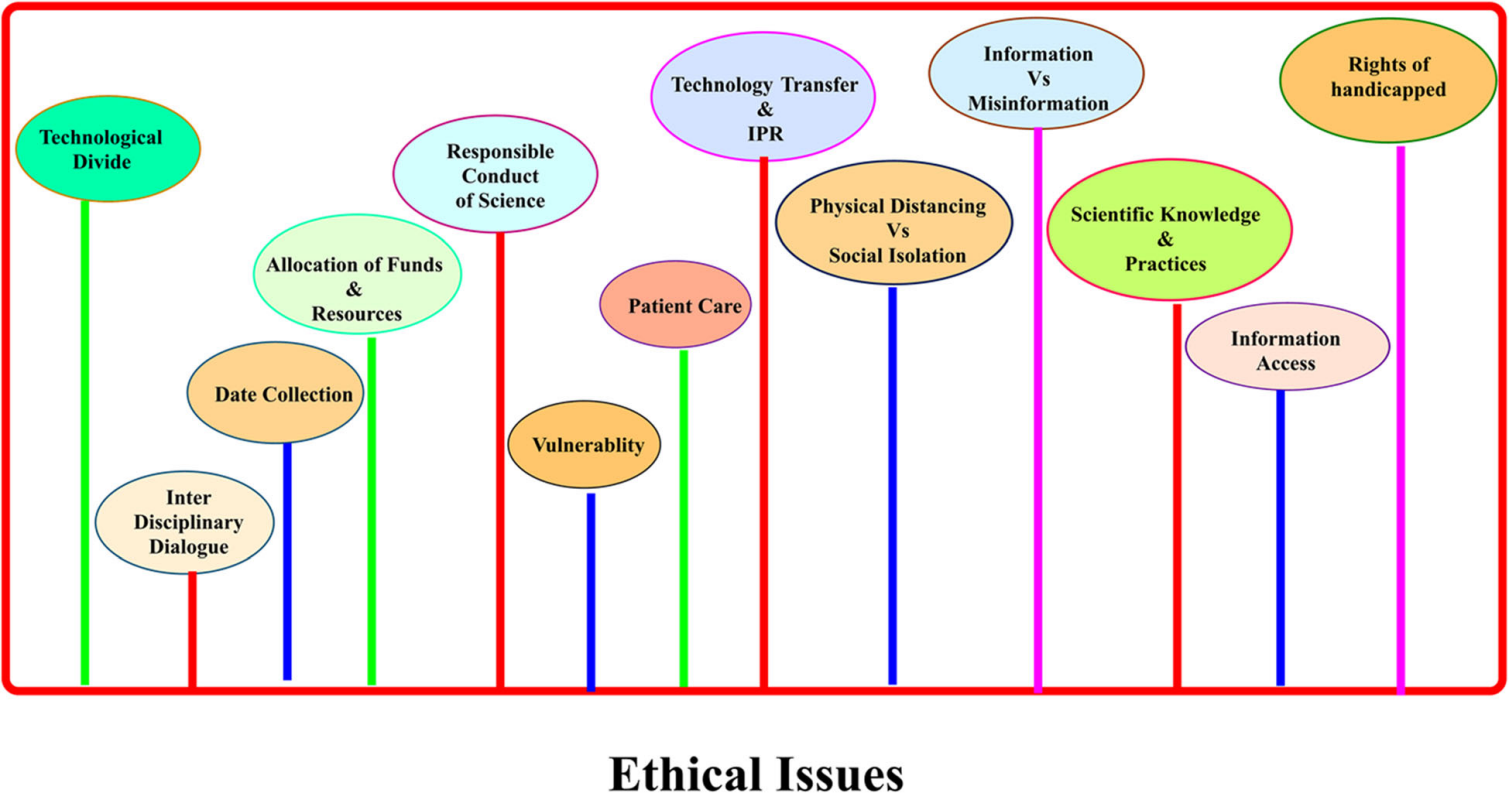
Ethical issues arising during the current COVID-19 pandemic

Pandemic response should be guided by the ethical principles of fairness, respect and transparency. However, outbreaks are more often confronted with fear, discrimination, and interventions lacking evidence which raises public health concerns [33]. In this section, we discuss the ethical challenges faced by low to middle income countries as they struggle to respond to the escalating spread of COVID-19. Based on the idea that no “one size fits all”, it is important to consider how the cultural and economic values in these countries impact approaches to address the corresponding ethical issues [34]. Figure 4 indicates the issues in the LMICs regarding global health emergencies using an ice berg analogy. Various ethical dilemmas arising from the current situation are indicated in Fig. 5.

### Preparedness challenges in LMICs

Rapidly growing contagion in less developed countries mainly in Africa, Asia and certain parts of the Americas is a global health emergency. Different countries require a context-specific response depending upon their current situation whether there are no cases, infrequent cases, clusters or local transmission. Overall, decisive actions necessitate effective social distancing, quarantines and if required even lock downs as well as massive testing and systematic contact tracing to stop further spread. Developing and least developed countries are the most vulnerable to this crisis, many of which are affected due to war conflicts, are overly populated with urban areas and slums, lack access to basic health services and are thus at high risk of COVID-19 spread [35].

In LMICs, the greatest challenge is how fast the gaps in early response to COVID-19 outbreak are filled before the infection control goes out of hand. The best chance is to have the systematic containment measures in place and massive testing done before the virus overwhelms the weaker health care systems. A well-organized response should also incorporate scientific knowledge generation e.g. studies on changing disease epidemiology such as duration of incubation period between infection and appearance of first symptoms so the people are retained in quarantine no longer than it is necessary in order to keep the costs down [36]. Moreover, rapid and actionable research conducted at local level should be encouraged in LMICs so as to deal with the pandemic more effectively. Data generated from response activities can be utilized for research purposes to make foreseeable predictions in the local context [37] and change the ongoing response strategies as and when required in order to minimize socio-economic impacts.

Response preparedness is weak in many low income countries as evident by preparedness assessments of 45 countries, none of which were evaluated as ready to respond, making them predominantly susceptible to epidemics. It is due to the poor health and nutrition conditions, aggravated by co-morbidities and low average annual health spending of only $267 per person in these countries. According to WHO, the regional readiness level is assessed to be only 66% with serious gaps in the response capacities for these countries to investigate disease spread alerts, treatment of patients in quarantine facilities and transmission control in both the health facilities and the public [38].

South Asia which holds a quarter of the world’s population with currently COVID-19 affected countries including Afghanistan, Pakistan, India, Nepal, Bangladesh, Sri Lanka are likely to face severe constraints in the management of the outbreak if it spreads uncontrollably. The current low number of reported cases may be due to less testing with limited resources in these countries. For example, India’s testing rate is exceptionally low given its large number of population with an average of just over 10 tests per million persons which is way less than advanced countries like South Korea with more than 5500 and Italy more than 2500 tests per million persons, as of March 17, 2020 [39]. Total cumulative corona cases in India were reported to be 4,754,356 while the death toll has risen to 78,586, as of 13th September 2020 [3]. Pakistan reported its first coronavirus case on February 26, 2020. There were 2880 confirmed cases and 45 deaths, as of April 5, 2020. The weekly report of 7th to 13th September 2020 by WHO reveals a total of 301,481 cumulative cases of SARS-CoV-2 in Pakistan, with 6379 cumulative deaths [3]. Initially, the country’s response was appropriate and timely just when the virus was already spreading from china to its neighboring countries due to travel. The containment measures proved effective in preventing the import of virus from China. Later, when a considerable number of people travelled back from neighboring Iran which was badly affected by the virus, the whole dynamics changed for Pakistan. Partial or complete lockdowns were imposed throughout the country, and all businesses apart from those providing essential goods were closed [40]. The government estimated that the number of cases were expected to rise up to 50,000 by April 25, 2020 in a national action plan report submitted to the Supreme Court [41, 42]. However, the lockdown situation was gradually eased with implementation of “Smart Lockdowns” and reopening of the economy in stages.

Afghanistan, a war-torn nation started to feel the brunt of COVID-19 just like its neighbors. Controlling its spread in Afghanistan is governed by a number of social and political complexities, including the incursion of Afghan refugees from neighboring Iran. Less public awareness of the virus and lower health literacy is a major issue illustrated by an individual who was confirmed to have the virus, and 37 people who were the potential suspects, left the quarantine facility, risking the virus transmission in the communities [43]. The cumulative deaths in Afghanistan have risen to 1420 while the total reported cases are 38,641 as of 13th Sept. 2020 [3]. Iran faced the worst situation among LMICs and was the epicenter of corona virus in Asia with over 50,000 confirmed cases and over 3000 deaths as of April 5, 2020. As of 13th Sept. 2020, the total number of reported cases are 399,490 and cumulative deaths are reported to be 23,029 in Iran [3]. The Iranian government was criticized for failure to respond early which resulted in shear increase in the number of cases, affecting both citizens and several top officials [44]. Also, insufficient public awareness regarding risk of the virus, and poor public attitude in observing self-quarantine were attributed as reasons for higher rate of spread [20]. In addition to resource limitations, US sanctions on Iran even increased the difficulty in procurement of medical supplies from companies abroad. It is due to the stricter sanctions imposed by US since May, 2019 with severe penalties for non-US firms doing business with Iran. This is a humanitarian crises and the global community must look at the impacts of such sanctions on humanitarian aid during a pandemic so that the sufferings of the public could be reduced [45].

Some countries also faced challenges in implementation of ongoing lockdowns due to religious or cultural values such as religious congregations [46]. Congregations in Pakistan, Malaysia and India were considered responsible for transmission of the virus. Pakistan reported hundreds of cases directly linked with the congregation which was held in March at Raiwind, Lahore [47]. Developing and less developed countries also face several challenges in self-quarantine which might not be very effective where large families live together often in congested settings, sometimes three or more people sharing the sleeping quarters. Households in Sierra Leone, Tajikistan, Guinea, Pakistan, Afghanistan, and Senegal are the largest, with six or more members on average [48].

In Africa, the first case was confirmed in Egypt on Feb 14, 2020. According to the recent data of WHO available on 13th Sept. 2020, the total number of cases has risen to 1,116,321 with the death toll rising to 23,916 in African continent, with South Africa affected the most [3]. The COVID-19 outbreak continues to spread across Africa with a number of countries in the continent where community transmission is becoming established such as South Africa. African countries are more vulnerable to faster spread of COVID-19 due to weak health care systems, high occurrence of HIV and malnourishment among other factors such as scarcity of medical supplies for personnel and the patients [49]. Resource constrained countries in Africa, should take steps for prepardeness and development of basic technological interventions for responding to health emergency [50]. The WHO African regional office along with CDC immediately started taking measures to prepare African countries for COVID-19 outbreak. The previous experiences from Ebola preparedness came handy as coordination response mechanisms were already in place. Over the past few years, the WHO has helped develop a national network of surveillance laboratories and health facilities in the African region amidst the previous outbreaks which could prove really helpful in current crisis [38]. The rapid response measures taken in China and other countries like Taiwan, Hong Kong, Singapore and South Korea ranged from strict quarantine measures, to detailed contact tracing, augmented with use of big data analytics. These measures helped the countries in keeping down the number of growing cases by breaking the chain of transmission. Taiwan leveraged all the technological resources, integrating national health insurance and immigration and customs databases to generate big data for tracing potential cases or areas [51]. The impacts of these early interventions for effective response towards COVID­19 is encouraging for the countries where COVID-19 is spreading fast.

### Ethical obligations with respect to public engagement

Effective public engagement should be made meaningful through gaining public trust and seeking cooperation instead of using the coercive measures especially in resource constrained settings with low level of literacy and social, religious and cultural complexities. Only this way, the lockdowns or quarantine measures will be more effective [52]. Effective risk communication is mandatory in public health response measures taken in LMICs ensuring the public’s right of access to information. Poor populations without access to information channels are the most vulnerable during health crisis and are most likely to ignore the government’s warnings regarding the precautionary measures such as social distancing [53]. A larger population of LMICs is living without access to mass media in rural areas or some poor countries such as Madagascar, Nigeria, Zambia etc. [48].

Awareness about the risk can be spread through simple health messaging and regular briefings by the government on television and radio; through public officials at the district level; or any other means deemed appropriate so as to provide access even to the poorer communities living without internet or communications channels. It is the government’s obligation to keep the public well informed about the risk of COVID-19. It also means that governments may have to take difficult decisions given the uncertainty and time constraints surrounding this pandemic. Therefore, it is important that information must be communicated in a transparent, honest and timely manner [51, 54].

### Ethical obligations with respect to resource allocation

Scarcity of resources including trained personnel, health centers, and protective gears is a major problem in LMICs. Even under normal circumstances, the poorest countries have acute shortage of ICU beds in comparison to high income countries e.g. roughly US has 33 ICU beds per 1 million people whereas countries such as India, Pakistan and Bangladesh have only 2 beds per 1 million people. The situation is worse in sub-Saharan Africa where Zambia has 0.6 ICU beds per million, Gambia has 0.4 and Uganda has 0.1 beds per million. So the fatality rate in these countries is estimated to be much higher in these countries than wealthier nations [55]. During a pandemic, standard crises care protocols should be developed by public health institutions to establish a systematic and evidence based procedure which ensures fair distribution of health care resources. Thus shifting the focus from prioritizing individual patient benefits to maximizing benefits to the community as a whole [56]. Priority decisions regarding resource allocation should not be discriminatory i.e. based on sex, race, religion, disability, wealth, citizenship, social status or connections [52]. Moreover, the ethical debate regarding allocation of resources in LMICs must take into consideration a wider context where critical care resources may already be scarce or non-existent even in a normal situation as compared to developed countries. In such conditions, ethical justification encompasses social justice governed with locally adapted global approaches [57].

### Ethical obligation to compensate

Ethical standards support the idea that state is responsible for compensating the public losses incurred upon them due to public health interventions such as the containment measures including social distancing, quarantine and isolation. This is particularly important for people residing in resource constrained regions. The state ought to make social policies with the aim to share some of their burdens and costs e.g. by protecting the employment rights of citizens [58], providing financial support to the poor and needy such as daily wagers who might suffer due to shutting down of several industries as a result of lock down orders. However, lack of resources could seriously defeat this argument of compensation in these countries which cannot even provide for the basic health care needs of the people [58]. In Pakistan, it was estimated that between 12.3 million to 18.5 million workers in various industries were at risk of losing their jobs. According to Human Rights Watch, the government must tend to the poorer workers who might be further pushed into poverty and it may dissuade them from voluntary quarantine necessary to contain the spread of the virus [59].

## Capacity building in LMICs through multilateral response strategy

A multi-lateral response by international community has been previously seen against similar threats posed in 2003 by Severe Acute Respiratory Syndrome (SARS), in 2009 by swine flu (H1N1), in 2012 by Middle East Respiratory Syndrome (MERS) and in 2014–16 by Ebola. All these crises were contained well via multilateralism and current crises of COVID-19 is yet to be further materialized by this strategy. The current COVID-19 crisis presents challenges that are beyond and above the earlier outbreaks, hence it deserves a well-established multilateral response. Any pandemic requires the weak links to be strengthened on individual basis i.e. at the hospital level as well as community basis, country basis and even globally. Therefore, it is the urgent need to shore up the health care systems in order to handle the current flood of cases as well as the future waves of the same or other related viruses.

Efforts for developing and supplying medical devices, diagnostic tools, vaccines, therapeutics, and other medical technologies for COVID-19 pandemic can be seen globally. Even though medical and scientific urgency are building, the medical technologies need to be tested efficiently, ethically and urgently with equitable availability to everyone around the globe. Therefore, a multi-lateral response strategy which can accelerate scientific discovery and technology development with ensured safety, efficacy and quality is essential. Further, there is need to coordinate the World Health Organization (WHO) for operational implementations. Technology pooling and benefit sharing as previously witnessed during influenza [60] and SARS epidemic [61] will not only save lives of millions of individuals by response acceleration to pandemics but will also encourage powerful administration of the global solidarity for the future epidemics.

## Sharing information (rapid information sharing mechanism)

To protect people against deadly infectious disease outbreaks, it is critical that scientists and governments rapidly share information about the pathogens that cause them. The genetic information of SARS-CoV-2 was shared immediately and openly [62] that accelerated the initial stages of diagnostic tests development and novel therapeutic compounds exploration. Likewise, many researchers immediately shared their research information via open source publication [63]. Scientist from different countries are sharing medical course and epidemiological data and collaborating for medical guidelines development in response to the current pandemic [64].

Such examples of sharing information and open science need be incorporated throughout research and development of COVID-19 medical technologies. Moreover, the scientific community need to share every progress, every success and even the negative data so the research can be continued with uppermost speed to obtain the best results. Some of the current and early research by pharmaceuticals, universities and medical device companies are funded by charities and governments. It is therefore imperative that such funding agreements mandate full data sharing, open source publishing and open collaboration following ethical guidelines regarding identity of subjects.

A data-sharing system needs to allow collaboration between stakeholders in the absence of pre-existing relationships and all collaborators must adhere to fundamental ethical principles of data use. Above all, it must ensure that people in all affected countries benefit from timely access to evidence-based interventions in emergencies.

## Intellectual property rights (IPR) and regulatory barriers

The multi-lateral response needs to be opened to the wide range of intellectual property rights, technology blueprints, technology specification, copyrights, patent rights, cell lines, research and regulation rights, data rights and clinical trial rights. In simple words, no exclusive right has to stand in the way of response to COVID-19 pandemic by global research community in order to prioritize public health. Similarly, all the rights such as confidential business information rights and trade secret rights required for bio similars, vaccine development and medical technology need to be accumulated and distributed hence to accelerate access to the market. It is therefore important to enable fast track registration along with emergency access to new medical technologies and medicines around the globe. Some of the regulatory barriers can be eliminated by access to regulatory data and fast-track registration, however, countries should ensure that producers can bring medical technologies quickly into the market with equitable access.

## Granting licenses can utilize all the available capacity of medical technologies

Competition between producers has always resulted in increased supply with lower prices. In response to current pandemic, facilitating competitive supply source can present more advantages. As coronavirus infection is exponentially increasing with life threating outcomes, there is need of utilizing every possible option to mobilize supply capacity with respect to diagnostics tests, therapeutics, protective equipment, vaccines and other medical devices as soon as possible. With expanding supply, necessary actions should be taken to limit the export of needed ingredients, medical technologies and hoarding of medical supply to other countries [65, 66]. Compulsory licensing is a useful tool to be used during public health emergencies such as the COVID-19 crisis when a treatment becomes available. International organizations and pharmaceutical companies should encourage the developing countries to pursue this option in the time of a pandemic [67].

## Conclusion

It would be difficult to minimize the socio economic impacts of COVID-19 in due time. It is the prime responsibility of the international community to take public health measures in best interest of the public with providing access to basic health care facilities, information and resources without discrimination, embodying the values of respectfulness and cultural appropriateness. In the long run, governments in developing countries should strive to achieve self-sufficiency through policy interventions by mobilizing local industries to manufacture medical care resources such as personal protective equipment, ventilators for capacity building and facilitate the propagation of scientific research and technological innovations. Once a vaccine is made available, it will be important to increase collaboration across the regions to ensure that the world’s poorest countries have an equal access to adequate vaccine supply. Through anthropogenic activities animal species are constantly under a severe threat of extinction amplified by the loss of biodiversity, global warming and animal trade. Global communities must unite for the climate action if we are to prevent any further global scale pandemics.

## Data Availability

Available on request.
